# The influence of a hot environment on parental cooperation of a ground-nesting shorebird, the Kentish plover *Charadrius alexandrinus*

**DOI:** 10.1186/1742-9994-7-1

**Published:** 2010-01-11

**Authors:** Monif AlRashidi, András Kosztolányi, Clemens Küpper, Innes C Cuthill, Salim Javed, Tamás Székely

**Affiliations:** 1 Department of Biology and Biochemistry, University of Bath, Bath BA2 7AY, UK; 2 Department of Ethology, Eötvös Loránd University, Budapest, Pázmány Péter sétány 1/c, H-1117, Hungary; 3 Centre for Behavioural Biology, School of Biological Sciences, University of Bristol, Woodland Road, Bristol BS8 1UG, UK; 4 Terrestrial Environment Research Center, Environment Agency, Abu Dhabi, PO Box 45553, United Arab Emirates

## Abstract

**Background:**

Parental care often increases offspring survival, but is costly to the parents. A trade-off between the cost and benefit of care is expected, so that when care provisioning by both parents is essential for the success of young, for instance in extremely cold or hot environments, the parents should rear their young together. We investigated the latter hypothesis in a ground nesting shorebird, the Kentish plover *Charadrius alexandrinus *in an extremely hot environment, the Arabian Desert. Midday ground temperature was often above 50°C in our study site in Abu Dhabi (United Arab Emirates), thus leaving the eggs unattended even for a few minute risks overheating and death of embryos.

**Results:**

Through the use of video surveillance systems we recorded incubation routines of male and female Kentish plovers at 28 nests over a full day (24 h). We show that ambient temperature had a significant influence on incubation behaviour of both sexes, and the relationships are often non-linear. Coordinated incubation between parents was particularly strong in midday with incubation shared approximately equally between the male and the female. The enhanced biparental incubation was due to males increasing their nest attendance with ambient temperature.

**Conclusions:**

Our results suggest biparental care is essential during incubation in the Kentish plover in extremely hot environments. Shared incubation may also help the parents to cope with heat stress themselves: they can relieve each other frequently from incubation duties. We suggest that once the eggs have hatched the risks associated with hot temperature are reduced: the chicks become mobile, and they gradually develop thermoregulation. When biparental care of young is no longer essential one parent may desert the family. The relaxed demand of the offspring may contribute to the diverse breeding systems exhibited by many shorebirds.

## Background

Biparental care of eggs or young is uncommon in the animal kingdom although it does occur among insects, fishes, amphibians, birds and mammals [1-4]. Biparental care, however, is a common behaviour in certain groups of animals: for instance 40% of cichlid fish genera and 32% of primate species are biparental [5]. These species provide excellent opportunities to investigate *how *and *why *biparental care evolves, and to tease apart the roles of parental investment, sexual selection and conflicts in breeding system evolution [*sensu*-[6-9]].

Two major groups of hypotheses have been proposed to explain biparental care [reviewed by [2,10,4]]. On the one hand, both parents may be essential for successful rearing of the young; the parents may need to share incubation, brood defence or protection of the territory in order for the young to survive [11,12]. Biparental care may be essential if parents breed in resource poor environments, or the physical environment is harsh and challenging [13]. Experimental removal of one parent (usually, the male) supports the hypothesis that biparental care provides direct benefits by enhancing offspring survival, and/or by putting less strain on the female [2,14-17]. On the other hand, parents may benefit in future from staying together and sharing care provisioning [18]; for instance by keeping their partner for future matings and therefore avoiding the costs related to finding/attracting a new mate. Staying with the mate and helping him/her might be particularly beneficial if there are few opportunities for finding a new mate [4].

Biparental care is particularly common among birds: approximately 50% of bird species have biparental incubation and/or brood care [11,12,19,20]. Although in these species the parents cooperate to rear the young, there are also elements of conflict because the benefit of care, i.e. the offspring, is shared between biological parents whereas each parent pays the cost of care itself. Therefore each parent prefers the other to invest more resources in rearing the young [sexual conflict over care [21,7,23]].

Incubation is essential for successful reproduction in nearly all bird species, because eggs require heat for embryonic development, and the incubating parent can defend the clutch from potential predators [24,19]. However, incubation can be costly to the parents because it demands time and energy, and the incubating parents themselves become exposed to predators [25,26]. By sharing incubation, the parents reduce the costs of time, energy and predation risk imposed upon them [19].

The optimal temperature for embryo development in most birds is between 36°C and 40.5°C, and if ambient temperature deviates from the optimum, parents regulate nest temperature by warming or cooling the eggs [27,28]. Overheating and chilling (hyper- or hypothermia, respectively) reduce egg survival and may cause nest failure. Hyperthermia is more harmful than hypothermia, since hot temperatures induce embryonic mortality faster than cold ones; embryos may survive 0°C for a short time period, whereas no avian embryo survives above 44°C [27]. Therefore parental care in hot environments, especially of ground-nesting birds where the eggs might be directly exposed to the heat of the sun, plays a vital role in preventing eggs from overheating [29,30].

We investigated parental cooperation - defined here as mutually beneficial interactions between the parents to maximise their reproductive success [31] - in a small cosmopolitan ground-nesting shorebird, the Kentish plover, *Charadrius alexandrinus *(body mass approximately 42 g), which breeds in temperate and subtropical environments [32-34]. Nests are sparsely filled with material such as straw, pebbles, mollusc shells and algae which may act as insulation materials to help regulate egg temperature [32,35]. Both parents participate in incubation: females usually incubate in the daytime whereas males incubate during night [36,37], although after hatching of the eggs one parent (usually the female) may desert the brood. The Kentish plover is an ideal species for studying parental behaviour, since it has variable parental care both within and between populations. Monogamy, polygyny and polyandry may all occur along with male-only, female-only and biparental brood care within a single population [38-41]. All three types of brood care that occur in Kentish plovers were recorded in the Arabian Desert, although biparental care of young appears more common than in temperate zone populations such as Hungary and France [42]. The transition from biparental incubation to biparental/uniparental brood care is an excellent paradigm to understand how and why animals shift from biparental care to uniparental one.

Here we investigate the division of parental effort during biparental incubation in Kentish plovers in the Arabian Peninsula where ground temperatures may exceed 60°C at midday during the breeding season. The objectives of our study were to answer two questions: i) Is the behaviour of the male or the female influenced by ambient temperature? ii) Does ambient temperature influence parental cooperation during incubation? We predicted that (i) male contribution to incubation should increase with ambient temperature to assist female incubation, and (ii) total incubation will increase with ambient temperature, so that parental coordination will be tight during the hottest part of the day.

## Methods

### Study area

Fieldwork was carried out in Al Wathba Wetland Reserve between 13^th ^of March and 23^rd ^of July 2005, and between 26^th ^of April and 12^th ^of July 2006. This reserve is located approximately 40 km south-east of Abu Dhabi in the United Arab Emirates (24° 15.5' N, 54° 36.2' E). The fenced reserve with a total size of about 465 ha is composed of artificially created water bodies that are surrounded by sand dunes. About 200 pairs of Kentish plover breed within and around the reserve [42].

### Data collection

Kentish plovers are sexually dimorphic during the breeding season which allows identification of sexes from photos [43]; adult males have black eye-stripes; black frontal head bars and incomplete black breast-bands, whereas these areas are pale brown in adult females. Sexual dimorphism in plumage fades over the season, therefore at four nests in 2005 the eye and head stripe of males were dyed using black permanent marker to facilitate discrimination between males and females from the nest photos.

**Figure 1:**
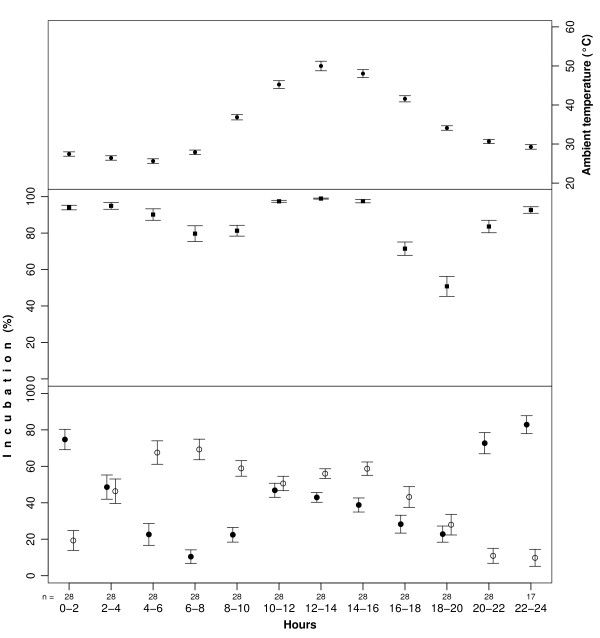
**Ambient temperature (top panel), and incubation by male (filled circles), female (opened circles) and both parents (filled squares) at Kentish plover nests in Abu Dhabi (mean ± SE for each two-hour time period, n is the number of nests used in the analyses)**.

Activities at the nests were recorded using a small spy camera (Outdoorcam, Swann Communications Pty. Ltd.) positioned about 1 m from the nest [44]. The camera was connected to a digital video recorder (MemoCam, Video Domain Technologies Ltd.) that recorded an image every 20 s. The camera was equipped with infrared lights to capture the images of incubating plovers during night. Power was supplied by a car battery (12 V). All parts of the system (except the camera), and the cables were hidden underground to minimize the disturbance to the birds. The ambient temperature was measured by a thermo-probe which was placed about 25 cm from the nest scrape at ground level. The probe was connected to a data logger (Tinytag, Gemini Data Loggers Ltd.) that recorded the temperature every 20 s. Ground temperature often exceeded 50°C at midday (Fig. 1); the maximum ground temperature recorded was 64.8°C.

To control for seasonal changes, we noted egg-laying date (the date of the last egg laid in the clutch) or estimated it by floating eggs in lukewarm water [45]. Egg-laying was then calculated as the number of days between 1 March and egg-laying date.

Ambivalent identification of male and female, and records when the parents were disturbed (e.g. during daily function tests of the data recording systems) were excluded from analyses (< 2.3% of all records). At 11 nests data between 22:00 h and 24:00 h were not used in the current analyses, because these nests were manipulated after 22:00 h [see [44]]. In total, data from 28 nests (20 and 8 nests from 2005 and 2006, respectively) were included in the analyses.

### Statistical procedures

Daily 24 h recordings starting at midnight (0.00 h) were considered as the unit of analysis. Each day was divided into twelve 2 h time periods. Five behavioural variables were calculated for each interval: (1) *total incubation*, the percentage of time when the eggs were incubated by either parent; (2) *male incubation*, the percentage of time when the eggs were incubated by the male, (3) *female incubation*, the percentage of time when the clutch was incubated by the female, (4) *changeovers*, the number of cases when one parent was relieved by the other parent, (5) *length of off-nest periods*, the mean of off-nest periods that began in the given interval. For variables (1)-(3) an equivalent terminology would be 'nest attentiveness', although to be consistent with our previous studies we prefer to use the term incubation [46]. Note that by incubation we mean keeping the egg temperatures close to the optimal temperature for embryonic development, and thus it includes both keeping the eggs warm and shading them for excessive ambient heat. Proportion variables were arcsine square-root transformed for normality. Changeovers and length of off-nest periods were ln(*x *+ 1) transformed for normality. The average ground temperature measured during each period was taken as the ambient temperature.

The influence of ambient temperature on incubation behaviour was investigated using linear mixed-effects models [47] with nest identity as a random factor, since parental behaviour is not independent between two-hour time periods for a given nest. Year, egg laying date and nest age (i.e. the number of days since the start of incubation) potentially influence incubation behaviour. We tested all three covariates on our response variables (total incubation, male incubation, female incubation, changeovers, length of off-nest periods), and the only significant effect was a difference in length of off-nest periods between years (mixed-effects models *P *= 0.020, all other *P *≥ 0.060), therefore these variables were not included in further analyses. However, for length of off-nest periods we checked that our conclusion does not change if we include the significant year effect in the final model (Table 1).

**Table 1:** Final mixed-effects models of incubation behaviour in the Kentish plover (Type III SS ANOVA).

**Explanatory variables**	**Response variable**														
	
	**% total incubation** **df_error _= 262**			**% male incubation** **df_error _= 250**			**% female incubation** **df_error _= 261**			**Changeovers** **df_error _= 262**			**Length of off-nest periods** **df_error _= 208**		
	
	**df**	***F***	***P***	**df**	***F***	***P***	**df**	***F***	***P***	**df**	***F***	***P***	**df**	***F *(*F*)**	***P *(*P*)**
	
**Time period**	11	1.44	0.157	*11*	*2.61*	*0.004*	11	0.81	0.632	11	1.138	0.332	*11*	*1.980 (2.019)*	*0.032 (0.028)*
**Ambient temperature**	2	0.25	0.782	2	0.35	0.704	2	0.75	0.475	2	0.856	0.426	2	0.877 (0.773)	0.418 (0.463)
**Female incubation**				*1*	*118.21*	*<0.001*									
**Male incubation**							*1*	*614.79*	*<0.001*						
**Time period × ambient temperature**	*22*	*3.25*	*<0.001*	*22*	*2.95*	*<0.001*	*22*	*2.51*	*<0.001*	*22*	*1.971*	*0.007*	*22*	*1.744 (1.733)*	*0.024 (0.026)*
**Time period × female incubation**				*11*	*3.36*	*<0.001*									

Ambient temperature was included in the models as second degree orthogonal polynomial. Statistically significant terms are in italics. *F *and *P *values in parentheses are taken from the alternative model that included also the significant (*P *= 0.037) year effect.

The initial models of total incubation, changeovers and length of off-nest periods included time period as a fixed factor and ambient temperature as second degree orthogonal polynomial covariate because avian incubation behaviour and ambient temperature are not linearly associated [28], and the interaction term between time period and ambient temperature. Our initial model for male and female incubation included time period (fixed factor), ambient temperature (second degree orthogonal polynomial covariate), the incubation by the other sex (covariate) and all second-order interactions. All models included a random intercept term for each nest. The initial models were fitted using maximum likelihood method, and model selection was carried out using the function stepAIC [48]. The final models were refitted using restricted maximum likelihood (Table 1).

We checked whether the effects of temperature and temperature^2 ^on incubation behaviour is due to within-subject or between-subject effects using within-group centering [49,50], and concluded that there is no difference between the within-subject and between-subject effects in any of the response variables (*P *> 0.3). Therefore our results from mixed-effects models reflect the within-subject effects.

We illustrate the results of the final mixed-effects models on S1-S3 (additional files 1, 2, 3) by fitting mixed-effects models of the variables showed in the figures, and present the back-transformed fitted values and the observed data. We used R version 2.7.1. and 2.8.1. for statistical analyses. Values are given as mean ± SE unless stated otherwise.

## Results

### Daily routine

Overall, the mean total incubation was 85.7 ± 1.1% over the full day (n = 28 nests). Females attended the nest 44.5 ± 1.7% of time, whereas males attended the nest 41.3 ± 1.6% of time. Male and female incubation routines were different: females incubated the eggs mostly in morning and males in the evening and at night (Fig. 1). At night (20:00 - 6:00 h) total incubation was 90.8 ± 1.4%, with females and males spending 33.2 ± 3.6% and 57.6 ± 3.6% of their time respectively. In contrast, during daytime (6:00 - 20:00 h) the nests were attended 82.4 ± 1.4% of the time, with females and males attending 52.1 ± 2.1% and 30.4 ± 1.8% of their time respectively. The nest was attended by either parent over 70% of time in each period except 18.00-20.00 h, and the highest attendance was during midday (Fig. 1).

### The influence of ambient temperature on incubation

**Figure 2:**
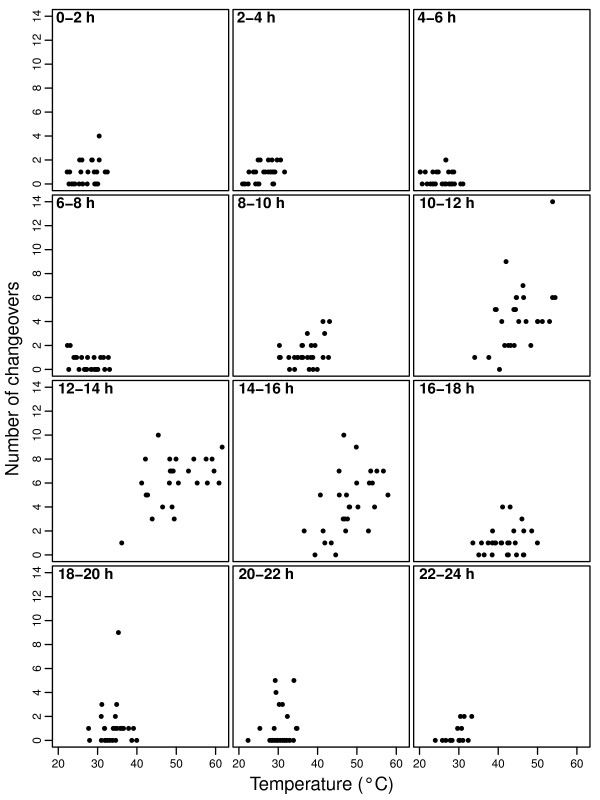
**Number of changeovers in relation to ambient temperature (°C)**.

**Figure 3:**
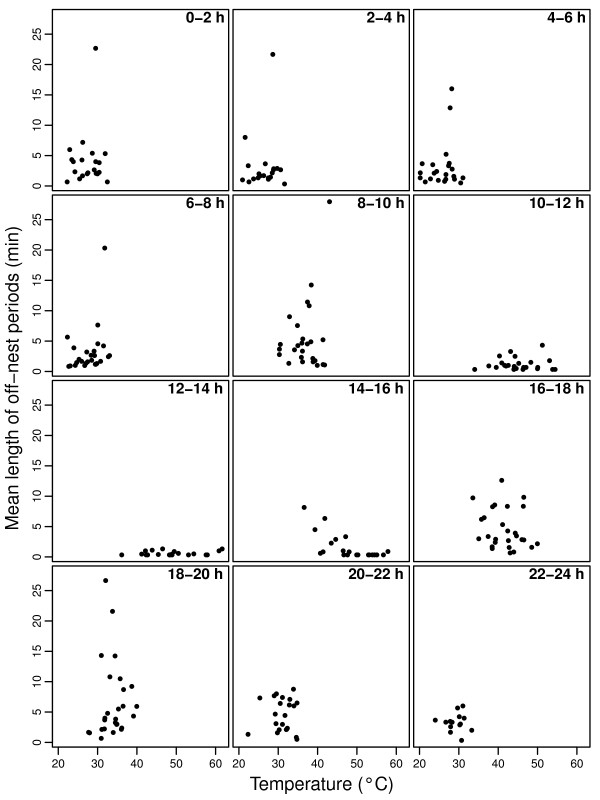
**Length of off-nest periods (mean length, in minutes) in relation to ambient temperature (°C)**.

Ambient temperature influenced incubation behaviour as with increasing temperature the parents changed over incubation more frequently (Fig. 2, Table 1), and the length of off-nest periods were reduced (Fig. 3, Table 1).

The effects of ambient temperature on incubation, however, were often not linear (Fig. S1-S3, additional files 1, 2, 3, Table 1). In the morning as ambient temperature increased, total incubation decreased with temperature. In midday, however, total incubation increased with temperature (Fig. S1, additional file 1). In late evening, total incubation decreased again with temperature.

Males and females responded differentially to ambient temperature during different parts of the day, as indicated by the highly significant interaction terms between time period and temperature (Table 1). During midday males usually increased incubation with temperature, whereas females decreased (Figs. S2-S3, additional files 2, 3).

### The influence of ambient temperature on parental coordination

**Figure 4:**
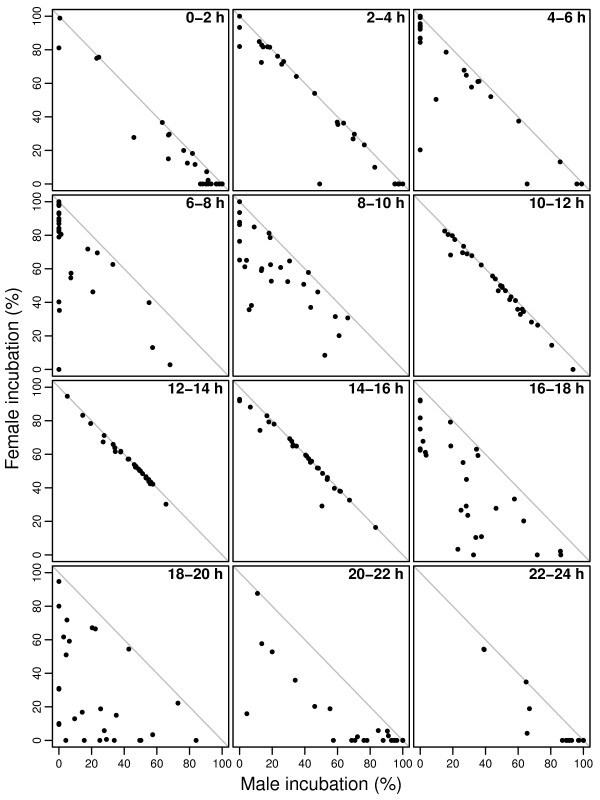
**Female incubation (%) and male incubation (%) in two-hour periods**. The grey line (-1 slope) shows the 100% total incubation threshold.

Female and male incubation tended to show a trade-off, and the strength of this relationship varied significantly over the day (Table 1, Fig. 4). In the morning (6:00 - 10:00 h) and in the evening (16:00 - 20:00 h) the inverse relationship between male and female incubation was poor. In contrast, during midday (10:00 - 14:00 h) the parents practically covered the nest continuously, and at most of the nests incubation was split approximately half between the male and the female (see also Fig. 1).

## Discussion

Our study provided two major results. Firstly, ambient temperature had a significant influence on incubation behaviour; the relationship was non-linear and depended on the time of the day. This suggests a complex relationship between incubation, ambient environment and time of the day. Incubation behaviour is expected to decrease with ambient temperatures until it reaches the optimal egg temperature and increase above the optimal egg temperature. However, as Figs S2-S3 (additional files 2, 3) suggest, there are many deviations from this pattern. The high resolution of our data taken over a full day, and *a priori *inclusion of time and ambient temperature effects, allowed us to reveal patterns that vary between different times of the day.

Secondly, understanding how a single parent responds to changing ambient conditions is not satisfactory, because in shared incubation systems the behaviour of a parent is influenced by the behaviour of its mate [51]. This is illustrated by the reversed response of males and females to ambient temperature during midday: male incubation tended to increase with temperature whereas female incubation was tended to decrease. In addition, the extreme temperature has important implications on parental coordination since parents showed tighter coupling in their incubation behaviour at high daytime temperature by sharing incubation equally and increasing number of changeovers. Following this strategy helps the parents coping with heat stress; they can relieve each other frequently from incubation duties.

Our results are in line with those of Purdue [32] who reported that nest attendance in the snowy plover *Charadrius alexandrinus nivosus *increased during hot parts of the day and off-nest periods were reduced. In late afternoon, however, nest attendance decreased, possibly because the latter period was suitable for foraging, and ambient temperature did not harm the embryos in the unattended eggs. Our results are consistent with the work of Amat and Masero [34] who suggested that hot ambient temperature may limit the length of incubation bouts, since females cannot sustain incubation for long periods. Hence increased incubation by the male in midday appears to assist the female in taking short time periods off the nest. However, we believe that our study goes beyond previous studies and is novel for several reasons. First, we used longer continuous records (24 h) and larger sample sizes. Second, we used linear mixed-effects models to control for non-independence between time periods, and statistically control for the effect of the other sex whilst investigating ambient temperature on the behaviour of the focal parent. Since incubation behaviour is usually not linearly associated with ambient temperature [28], we also use a quadratic term in our models. Finally, our conceptual framework was to understand how ambient environment influences biparental cooperation, whereas the focus of previous studies was the ability of a single parent to cope with heat stress.

An alternative explanation for the highly coordinated pattern of incubation is complex interactions (or negotiations) between parents [52] and coercion; for instance one parent may coerce the other to work harder. From the still images we took at the nests we cannot infer coercion, so that additional observational or experimental data are required to determine whether the female drives the precise timing of changeovers. However, we argue female coercion is unlikely. Male and female plovers have similar body sizes, and both sexes are well equipped for fighting [53]. In addition, coercion tends to be a trait of the behaviourally dominant sex, in this case, the male.

Results of our study are important for three reasons. Firstly, they suggest that at extreme hot temperature cooperation between the male and female parents is essential to raise the young. Parental behaviours, including incubation, provide good model systems to understand how two, usually unrelated, individuals cooperate in nature, given that the survival of their young often depends on care provisioning by both parents [7,4]. Secondly, incubation of ground-nesting birds puts the adults under severe heat stress in deserts, and interrupting incubation for more than a few minutes would kill the embryos [34]. These conditions should favour tight cooperation; the adults thereby reduce risk both to themselves and to their eggs. Males may be forced (in evolutionary time) to participate in daytime incubation, because females cannot manage the task alone. Therefore, extreme temperatures may increase the level of parental cooperation, and reduce sexual conflict over care [this study, [34]].

Thirdly, once the eggs have hatched the risks associated with high ambient temperature are reduced, since the chicks become mobile, and both the adults and their young can better regulate their body temperature, for instance by bathing more frequently or by moving under shade. Since chicks require a diminishing amount of protection, shading and attendance from their parents [23], desertion by one of the parents becomes less costly. Nevertheless, biparental care and parental cooperation may still be favoured by certain environmental variables such as localised food distribution and high predation on the chicks. For instance, when food distribution was patchy but abundant, the density of plovers increased and competition between families intensified [53], the parents spent more time defending their young and an extended biparental brood care was observed. In another study, Fraga and Amat [36] noted that long biparental care was a response to heavy chick predation in Kentish plovers by gull-billed terns *Sterna nilotica*, whereby protection by both parents were likely more effective than by a single parent.

## Conclusions

Our results suggest that extremely hot environment favours cooperation between incubating Kentish plover parents. The increased parental cooperation is essential, since a single parent cannot protect the eggs and/or itself from overheating. Experimental analyses of male-female interactions, and comparing the incubation responses of males and females across different plover populations are important avenues for revealing the complex relationships between ambient environment, parental cooperation and sexual conflict.

## Competing interests

The authors declare that they have no competing interests.

## Authors' contributions

ICC and TS conceived the study, and together with AK designed the project. Fieldwork was carried out by AK and CK. SJ provided the permissions and logistics. Statistical analyses were carried out by MAR and AK, and writing up was lead by MAR. The manuscript received inputs from all co-authors. All authors read and approved the final manuscript.

## Supplementary Material

Additional file 1 **Fig. S1 **Total incubation (%) in relation to ambient temperature (°C) in two-hour periods: observed (filled circles) and fitted values (asterisks) are shown from mixed-effects model (see Methods for details). Click here for file

Additional file 2 **Fig. S2 **Male incubation (%) in relation to ambient temperature (°C) in two-hour periods: observed (filled circles) and fitted values (asterisks) are shown from mixed-effects model (see Methods for details). Click here for file

Additional file 3 **Fig. S3 **Female incubation (%) in relation to ambient temperature (°C) in two-hour periods: observed (filled circles) and fitted values (asterisks) are shown from mixed-effects model (see Methods for details). Click here for file
